# *Glycyrrhiza glabra*-Based Nasal Spray as a Novel Treatment for Chronic Rhinosinusitis with Nasal Polyps: Efficacy in Symptom Reduction and Epithelial-Mesenchymal Transition Modulation

**DOI:** 10.7150/ijms.123281

**Published:** 2026-01-01

**Authors:** Geng-He Chang, Pey-Jium Chang, Yu-Ching Cheng, Ching-Yuan Wu, Yao-Hsu Yang, Yu-Shih Lin, Cheng-Ming Hsu, Ming-Shao Tsai, Yao-Te Tsai, Pei-Rung Yang

**Affiliations:** 1 Department of Otolaryngology, Chang Gung Memorial Hospital, Chiayi, Taiwan.; 2 Graduate Institute of Clinical Medical Sciences, College of Medicine, Chang Gung University, Taiwan.; 3 Faculty of Medicine, College of Medicine, Chang Gung University, Taoyuan, Taiwan.; 4 Head and Neck Infection Treatment Center, Chang Gung Memorial Hospital, Chiayi, Taiwan.; 5 Department of Traditional Chinese Medicine, Chang Gung Memorial Hospital, Chiayi, Taiwan.; 6 School of Traditional Chinese Medicine, College of Medicine, Chang Gung University, Taoyuan, Taiwan.; 7 Department of Pharmacy, Chiayi Chang Gung Memorial Hospital, Chiayi, Taiwan.

**Keywords:** CRSwNP, nasal spray, *Glycyrrhiza glabra*, epithelial-mesenchymal transition, histopathology, immunofluorescence

## Abstract

**Background:** Chronic rhinosinusitis with nasal polyps (CRSwNP) is a chronic inflammatory condition that significantly impacts patients' quality of life, characterized by high recurrence rates and limited treatment efficacy. Standard therapies, including corticosteroid nasal sprays, saline irrigation, and endoscopic sinus surgery, often fail to provide sustained relief, highlighting the need for alternative therapeutic strategies. *Glycyrrhiza glabra* (*G. glabra*), a traditional herbal remedy with documented anti-inflammatory and immunomodulatory effects, has shown potential in modulating inflammation in various respiratory and inflammatory conditions. This study evaluates the efficacy and mechanism of a novel *G. glabra*-based nasal spray for CRSwNP treatment, focusing on its anti-inflammatory and epithelial-mesenchymal transition (EMT)-reversing effects.

**Methods:** A total of 30 CRSwNP patients were enrolled and underwent a two-month single-regimen treatment with the *G. glabra* nasal spray without concomitant CRS medications, administered twice daily. Clinical outcomes were assessed via the Total Nasal Symptom Score (TNSS), Sinonasal Outcome Test-22 (SNOT-22), and nasal endoscopy. Histopathological evaluation was performed on nasal polyp biopsies from 15 patients pre- and post-treatment using immunohistochemistry (IHC) and immunofluorescence (IF) staining for mesenchymal and epithelial markers.

**Results:** Results demonstrated significant improvement in TNSS and SNOT-22 scores, alongside visible reduction in polyp size. IHC and IF analyses revealed reduced expression of mesenchymal markers (α-SMA, vimentin, fibronectin) and increased expression of epithelial markers (E-cadherin, EpCAM), indicating EMT reversal. No adverse effects were reported.

**Conclusion:** These findings suggest that* G. glabra* nasal spray may offer a safe and effective treatment option for CRSwNP, potentially acting through EMT modulation. Further studies with larger cohorts are recommended to refine patient stratification and explore the molecular basis of differential treatment responses.

## 1. Introduction

Chronic rhinosinusitis with nasal polyps (CRSwNP) is a prevalent and challenging inflammatory condition in otolaryngology, characterized by persistent inflammation, nasal obstruction, and a tendency for recurrence after treatment 1, 2. CRSwNP significantly impairs patients' quality of life, contributing to symptoms such as nasal congestion, reduced sense of smell, and facial pressure, which often require long-term therapeutic management 1, 2. Current standard treatments for CRSwNP primarily include intranasal corticosteroids, saline irrigation, and in more severe or refractory cases, endoscopic sinus surgery 3, 4. However, even with these treatments, recurrence rates remain high 1, 2, and some patients respond inadequately to local therapies, underscoring the need for novel, effective, and minimally invasive treatment options.

Emerging research has increasingly focused on the pathophysiological mechanisms underlying CRSwNP, particularly the role of epithelial-mesenchymal transition (EMT) in nasal polyp formation and persistence 5-8. EMT is a biological process whereby epithelial cells lose their characteristic markers and acquire mesenchymal features, enabling enhanced migratory capacity. This transition is believed to contribute to fibrosis and tissue remodeling within the nasal mucosa, processes that are instrumental in polyp development and chronic inflammation in CRSwNP 5-8. Specific biomarkers, such as E-cadherin and EpCAM for epithelial cells and vimentin and N-cadherin for mesenchymal cells, have been identified as markers of EMT activity within nasal polyps 5-8. Understanding and reversing EMT in CRSwNP could offer a promising therapeutic strategy for improving patient outcomes.

*Glycyrrhiza glabra* (*G. glabra*), a species of licorice plant and a traditional herbal remedy, contains glycyrrhizic acid as its main bioactive compound. It has documented anti-inflammatory and immunomodulatory effects and shows potential in modulating inflammation in various respiratory and inflammatory conditions, particularly rhinitis and sinusitis 9, 10. *G. glabra* extract, particularly in the form of nasal spray or irrigation, may offer an alternative approach by reducing inflammatory cytokine levels and inhibiting fibroblast activation, thereby potentially reversing EMT and decreasing nasal polyp size. Preliminary studies, including our previous work on allergic rhinitis and nasal polyp treatment 11, 12, suggest that *G. glabra* extract can mitigate nasal inflammation through mechanisms involving inhibition of the MAPK/Erk pathway in nasal polyp-derived fibroblasts. These findings highlight *G. glabra*'s potential as an adjunctive therapy in managing inflammatory nasal conditions.

To investigate the therapeutic potential of *G. glabra* nasal spray in treating CRSwNP, we initiated a clinical study at a single-center hospital setting, enrolling patients diagnosed with CRSwNP. This study employed a multi-faceted approach, combining subjective assessments through validated symptom questionnaires, objective evaluations using nasal endoscopy, and histopathological analyses of polyp biopsies to gain comprehensive insights into treatment effects. Our primary objective was to determine whether *G. glabra* nasal spray could effectively reduce symptom severity, decrease polyp size, and modulate cellular markers associated with EMT in CRSwNP. Through this study, we aim to explore *G. glabra* nasal spray as a novel, minimally invasive therapeutic approach with potential applications in managing this challenging condition.

## Materials and Methods

### *G. glabra* Nasal Spray Preparation

**Figure 1:**
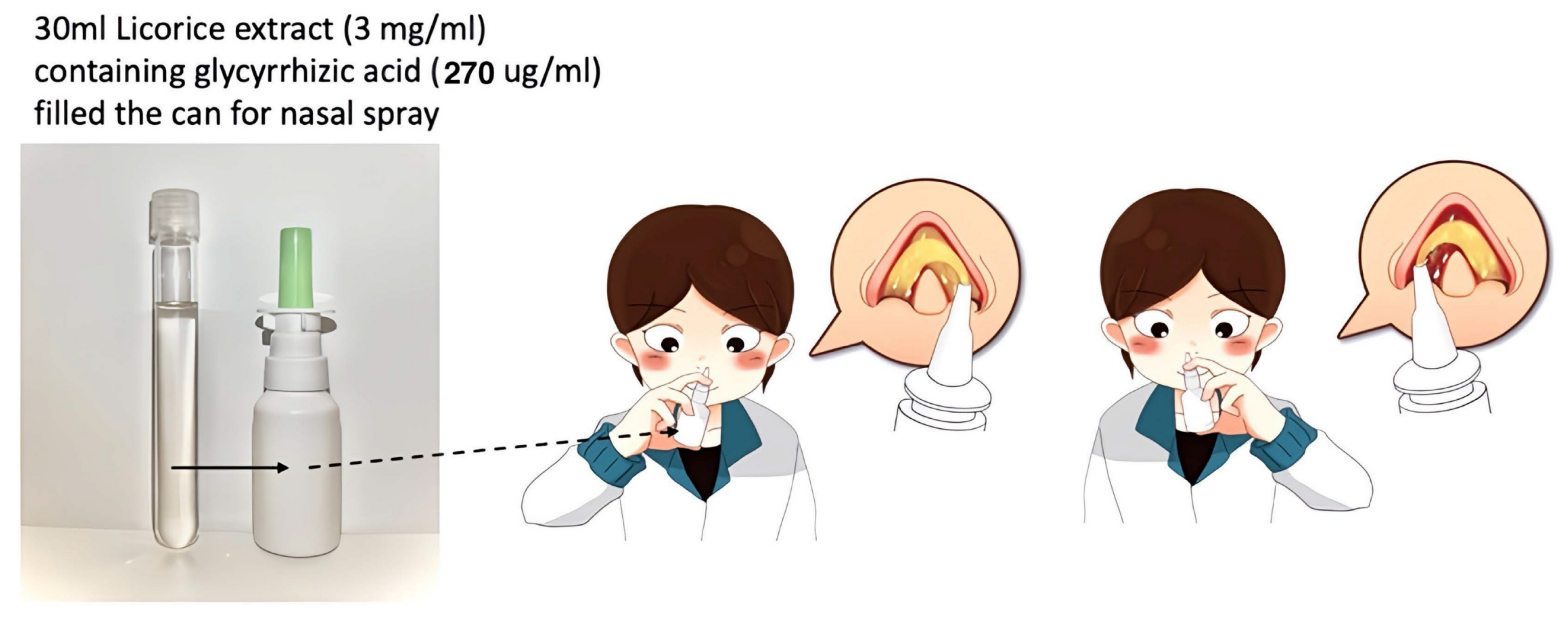
***G. glabra* nasal spray concentration and application.** This figure illustrates the *G. glabra* nasal spray used in the study, including its volume and concentration, with glycyrrhizic acid as the primary active compound. The person in the image demonstrates the application process, administering two sprays per nostril to ensure adequate delivery.

For this clinical trial, the* G. glabra* nasal spray was prepared following a standardized protocol to ensure consistency in concentration and therapeutic efficacy. The preparation began with 450 grams of dried *G. glabra* root slices, which were boiled in 11000 milliliters of distilled water for two hours to extract bioactive compounds, particularly glycyrrhizin. Slices of *G. glabra* root (sample number: No.7H-E014), obtained from Chung Ching Tang Chinese Herbal Medicine Pharmacy in Taipei and sourced from Ordos City, Hangjinqi, Inner Mongolia (GPS coordinates: 39.83119, 108.73507), were stored in the Department of Pharmacy at Chiayi Chang Gung Memorial Hospital. The boiled extract was then filtered to remove particulate matter and impurities. The resulting *G. glabra* solution was then freeze-dried to produce a concentrated *G. glabra* powder. 9 grams of freeze-dried *G. glabra* powder was dissolved in 300 milliliters of saline solution, achieving a final *G. glabra* concentration of 3 mg/mL 11, 12. This solution was then divided into 10 individual nasal spray bottles, each containing 30 mL of *G. glabra* nasal spray (Fig. 1).

High-performance liquid chromatography (HPLC) was employed to quantify the glycyrrhizin content within the solution, a key active component of *G. glabra* with anti-inflammatory properties. To meet the established therapeutic standards 11, 12, the glycyrrhizin content was required to exceed 20%, with a target concentration between 250 and 300 μg/mL. These parameters ensured that each batch conformed to the glycyrrhizin concentration range defined by previous studies 11, 12, providing a reliable basis for consistent dosing in clinical use.

### Clinical Trial

For this clinical trial, we aim to recruit 30 adult patients, aged 20 years and older, who have been diagnosed with CRSwNP based on established diagnostic criteria, including persistent nasal congestion, rhinorrhea, and nasal obstruction lasting over 12 weeks, along with evidence of nasal polyps confirmed through endoscopy or imaging 13. Eligible participants must be willing to adhere to the study protocol, which includes regular follow-up visits, symptom assessments, and nasal endoscopy. All participants will provide signed informed consent prior to enrollment in the study.

Patients are excluded from participation if they have a history of sinonasal or nasopharyngeal malignancy, or if they have recently used oral or intranasal steroids or antihistamines within the past month. However, those who discontinue these medications for a month may be reconsidered for inclusion. To ensure that this study was conducted as a single-regimen treatment, all enrolled patients were clearly instructed not to use any additional medications for CRS, including other nasal sprays, oral antihistamines, leukotriene modifiers, decongestants, or systemic steroids, throughout the entire 8-week treatment period. Concomitant medication use was reviewed at each visit, and adherence to this instruction was reinforced. Patients requiring rescue medications for acute exacerbations were to be withdrawn from the study analysis. Pregnant and lactating women are also excluded from the study to avoid potential risks associated with the treatment.

This clinical trial protocol has received ethical approval from the Institutional Review Board of Chang Gung Memorial Hospital (IRB approval number: 202202145A3).

### Study Protocol and Follow-Up

This study was conducted over a two-month treatment period, during which participants were instructed to use the *G. glabra* nasal spray twice daily, administering two sprays per nostril each morning and evening. Patients were scheduled for monthly follow-up visits, resulting in two evaluations over the course of the study. At each follow-up, participants completed subjective symptom assessments through validated questionnaires, while objective evaluations were conducted using nasal endoscopy to document any visible changes in polyp size and nasal mucosa status.

To provide insights into cellular-level changes, nasal polyp biopsies were performed at the beginning and end of the treatment period in patients who consented. These biopsy samples were used for histopathological and immunohistochemical analysis, allowing us to examine potential changes in inflammatory cell presence and EMT-related biomarker expression following *G. glabra* nasal spray treatment.

### Evaluation Methods

The study employed both subjective and objective assessment tools to evaluate treatment effects on CRSwNP symptoms and polyp characteristics. Subjective symptom severity was measured using the Total Nasal Symptom Score (TNSS) 11 and the Sino-Nasal Outcome Test-22 (SNOT-22) 11, 14, 15. The TNSS is a four-symptom scale (nasal obstruction, rhinorrhea, sneezing, and itching), with each symptom scored from 0 (no symptoms) to 3 (severe), resulting in a total score ranging from 0 to 12. The SNOT-22 includes 22 items related to nasal and sinus symptoms, each rated on a 0 to 5 scale, providing a comprehensive symptom impact score from 0 to 110.

The MLK score is an endoscopic grading system assessing three aspects: polyps, edema, and secretion 16, 17. Polyps are scored based on their presence in or beyond the middle meatus. A score of 0 indicates no polyps, 1 for polyps confined to the middle meatus, and 2 for polyps extending beyond the middle meatus. Edema is graded from 0 to 2, where 0 indicates no edema, 1 mild/moderate edema, and 2 severe edema. Secretions are scored from 0 to 2, with 0 for no secretion, 1 for serous discharge, and 2 for purulent discharge. Each of these parameters is assessed bilaterally, with a maximum possible MLK score of 12.

The nasal polyp score assesses the extent of polyp growth from 0 to 4 per side 18, 19. A score of 0 indicates no polyps, 1 for polyps limited to the middle meatus, 2 for polyps extending beyond the middle meatus without causing obstruction, 3 for polyps that either extend beyond the middle meatus and cause partial obstruction or do not extend beyond the middle meatus but involve the olfactory cleft, and 4 for complete obstruction of the nasal cavity. The total score is the sum of both sides, with a maximum possible score of 8.

In this study, nasal polyp biopsies were obtained from patients before and after the eight-week* G. glabra* nasal spray treatment. To evaluate the histopathological changes in nasal polyps, we performed analyses using hematoxylin and eosin (H&E) staining, immunohistochemistry (IHC), and immunofluorescence (IF). IHC and IF were used to assess specific markers, with α-SMA staining to evaluate fibroblast activity and fibronectin levels to gauge extracellular matrix (ECM) production within the nasal polyps 12, 20-23. These markers allowed us to observe treatment-induced changes in fibroblast activation and ECM remodeling. Following these analyses, we further examined epithelial and mesenchymal markers to evaluate EMT changes associated with treatment.

### Statistical Analysis

In this study, statistical analysis was conducted to evaluate the treatment effects on TNSS, SNOT-22, MLK, and nasal polyp scores in the 30 enrolled CRSwNP patients. Pre- and post-treatment scores were compared using paired Student's t-tests to assess significant differences.

In the H&E staining assessment, we applied the INHAND five-tier grading system 24, a commonly used pathological scoring method, to classify inflammatory cell infiltration severity. Scores ranged from 0 to 5: 0 for non-reactive tissue (< 1% positive cells), 1 for minimal (1-5%), 2 for mild (6-25%), 3 for moderate (26-50%), 4 for moderately severe (51-75%), and 5 for severe (> 75%). This system provided a structured evaluation of inflammation levels in nasal polyp tissue.

For IHC and IF analyses, we employed the IRS (Immunoreactivity Score) system, which combines cell positivity (quantity) and staining intensity to produce a composite score 25. Quantity was assessed as the percentage of positive cells, scored from 0 (no positive cells) to 4 (> 80% positive cells), while intensity was categorized from 0 (no color) to 3 (strongly positive). The combined IRS scores ranged from 0 to 12, with classifications of negative (0-1), mild (2-3), moderate (4-8), and strongly positive (9-12).

To determine the significance of treatment-related changes in these scores, we used paired Student's t-tests to evaluate pre- and post-treatment differences in histological and immunohistochemical markers. On graphical representations, statistical significance is indicated by * for p < 0.05, ** for p < 0.001, and *** for p < 0.0001, with p < 0.05 considered statistically significant.

## Results

### HPLC Analysis of *G. glabra* Nasal Spray

**Figure 2:**
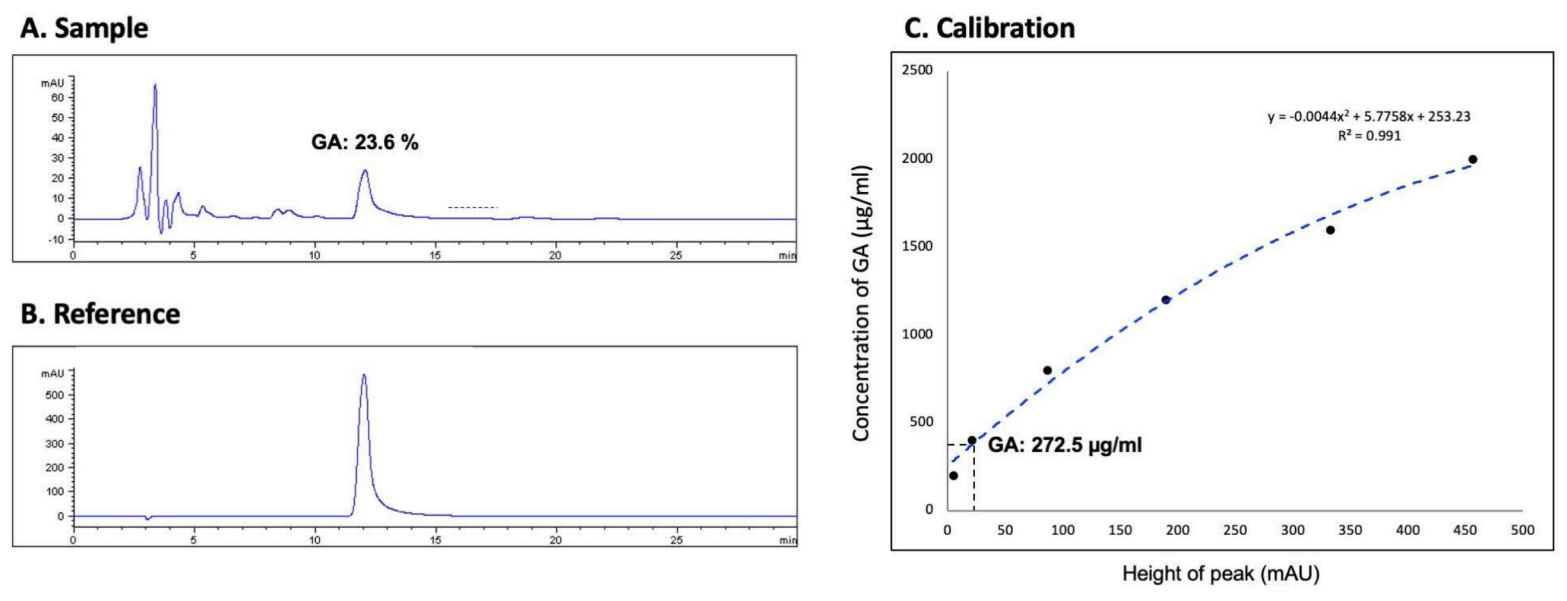
** HPLC analysis of glycyrrhizic acid concentration in *G. glabra* nasal spray.** HPLC was used to determine the glycyrrhizic acid (GA) content in the *G. glabra* nasal spray. (A) Chromatogram of the *G. glabra* nasal spray sample shows multiple peaks, each representing a different compound. The peak corresponding to GA is identified. (B) Chromatogram of pure GA used as a reference to confirm the GA peak in the sample. (C) Calibration curve generated from serial dilutions of GA, establishing a standard equation to calculate the GA concentration in the nasal spray.

Using HPLC on an Agilent 1100 HPLC system, we measured the glycyrrhizic acid (GA) concentration in the *G. glabra* solution used for nasal spray preparation to ensure standardization and consistency. The analysis was conducted with a Discovery® C18 column (15 cm × 4.6 mm, 5 µM), utilizing a mobile phase of 100% acetonitrile and 2% acetic acid at a 36:64 ratio. The flow rate was maintained at 0.6 mL/min, and the system was set to 25°C. Absorbance was monitored in the 250-360 nm range, and the GA concentration in the *G. glabra* nasal spray was determined to be 23.6%, with a final concentration of 272.5 µg/mL, as shown in Figure 2. A calibration curve was constructed to ensure accurate quantification, as shown in Figure 2C, with the sample results matched against a standard reference (Fig. 2A and 2B).

### Patient Demographics and Treatment Compliance

In this study, a total of 30 patients with CRSwNP completed the two-month treatment and follow-up period, and no patient required rescue medication or was excluded due to the use of additional CRS-related drugs. However, only 15 of these patients underwent nasal polyp biopsies both before and after treatment. Reasons for missing post-treatment biopsies included patient refusal and cases where the nasal polyps were too small for sampling. Throughout the treatment, none of the patients reported any adverse effects related to the *G. glabra* nasal spray. The demographic and baseline characteristics of the participants are summarized in Table 1. Among the 30 patients, 80% were male, with a mean age of 46.1 ± 12.8 years. Baseline scores for TNSS, SNOT-22, MLK, and nasal polyp scores were 6.73 ± 3.03, 41.2 ± 19.7, 9.20 ± 4.44, and 4.30 ± 1.62, respectively, indicating moderate to severe symptom and nasal polyp severity prior to treatment.

### Symptom Improvement in TNSS and SNOT-22

**Figure 3:**
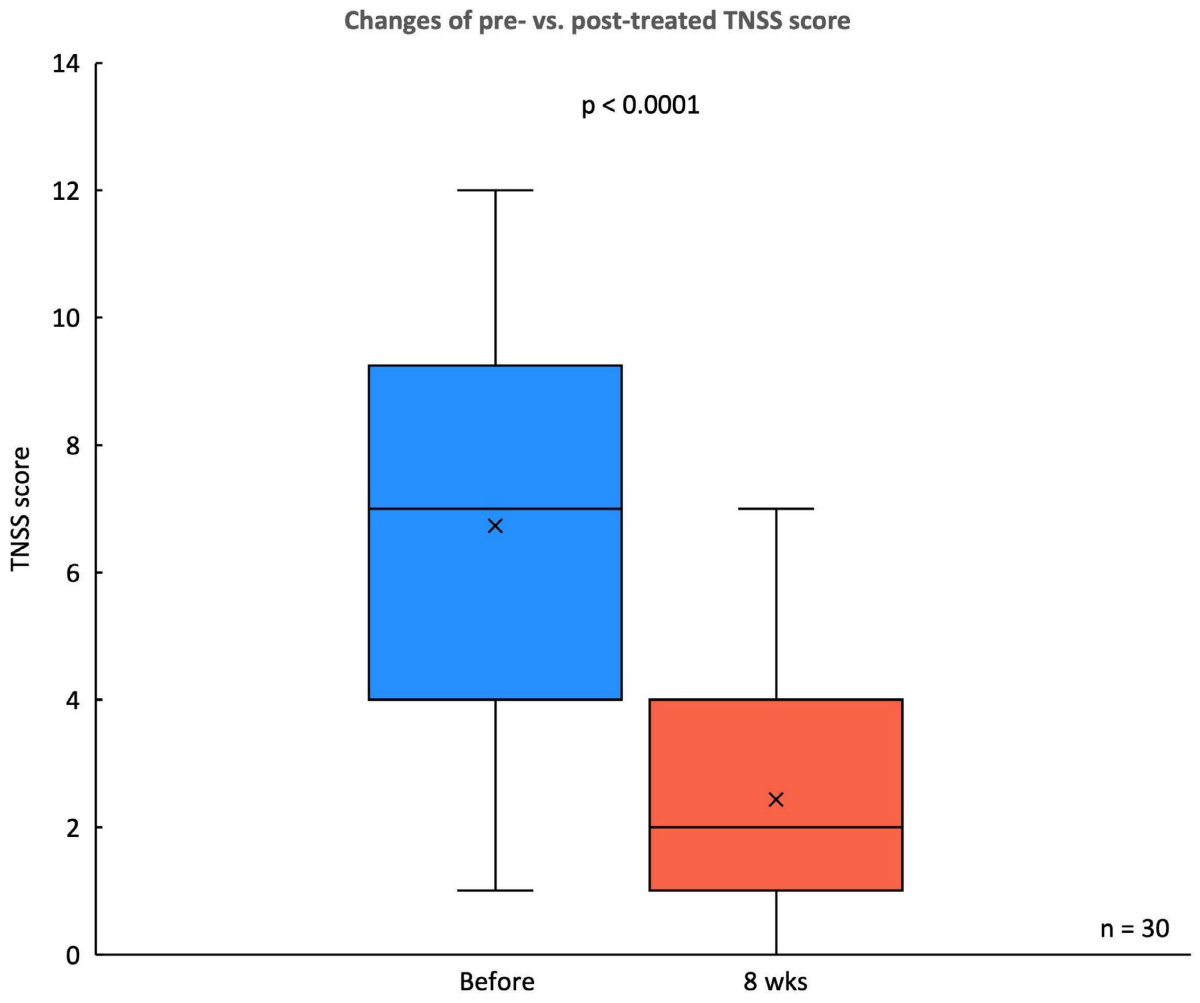
** Changes in TNSS before and after *G. glabra* nasal spray treatment.** Box plot comparing the TNSS scores of 30 patients with CRSwNP before and after 8 weeks of treatment with *G. glabra* nasal spray.

In this study, significantly subjective improvements in symptom severity were observed in the TNSS and SNOT-22 scores following an 8-week treatment period with the *G. glabra* nasal spray. The TNSS, a primary measure of nasal symptoms, showed a substantial reduction in mean score, decreasing from a pre-treatment score of 6.73 ± 3.02 to 2.43 ± 1.92 at 8 weeks (p < 0.0001) (Fig. 3). This indicates a marked alleviation of nasal congestion, rhinorrhea, sneezing, and itching among participants.

**Figure 4:**
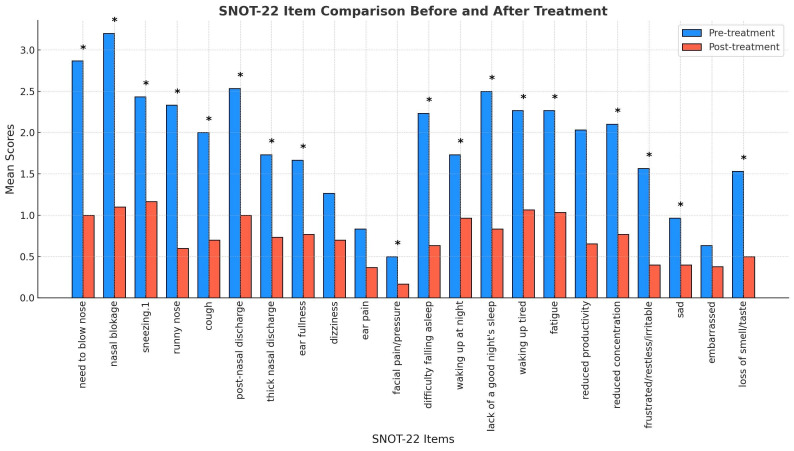
** Comparison of SNOT-22 scores before and after *G. glabra* nasal spray treatment.** Mean scores of individual SNOT-22 items comparing pre-treatment and post-treatment responses in 30 patients with CRSwNP. Items with significant improvements (p < 0.05) are marked with an asterisk (*), indicating notable symptom relief.

The SNOT-22 assessment, which evaluates a broader spectrum of symptoms related to CRS, demonstrated statistically significant reductions in 18 out of the 22 items (Fig. 4). Improvements were particularly evident in nasal-specific symptoms such as the need to blow the nose, nasal blockage, and thick nasal discharge, all of which showed a notable decline post-treatment (p < 0.05). Ear-related symptoms, however, such as ear dizziness and ear pain, did not reach statistical significance.

### Endoscopic Evaluation of MLK and Nasal Polyp Scores

**Figure 5:**
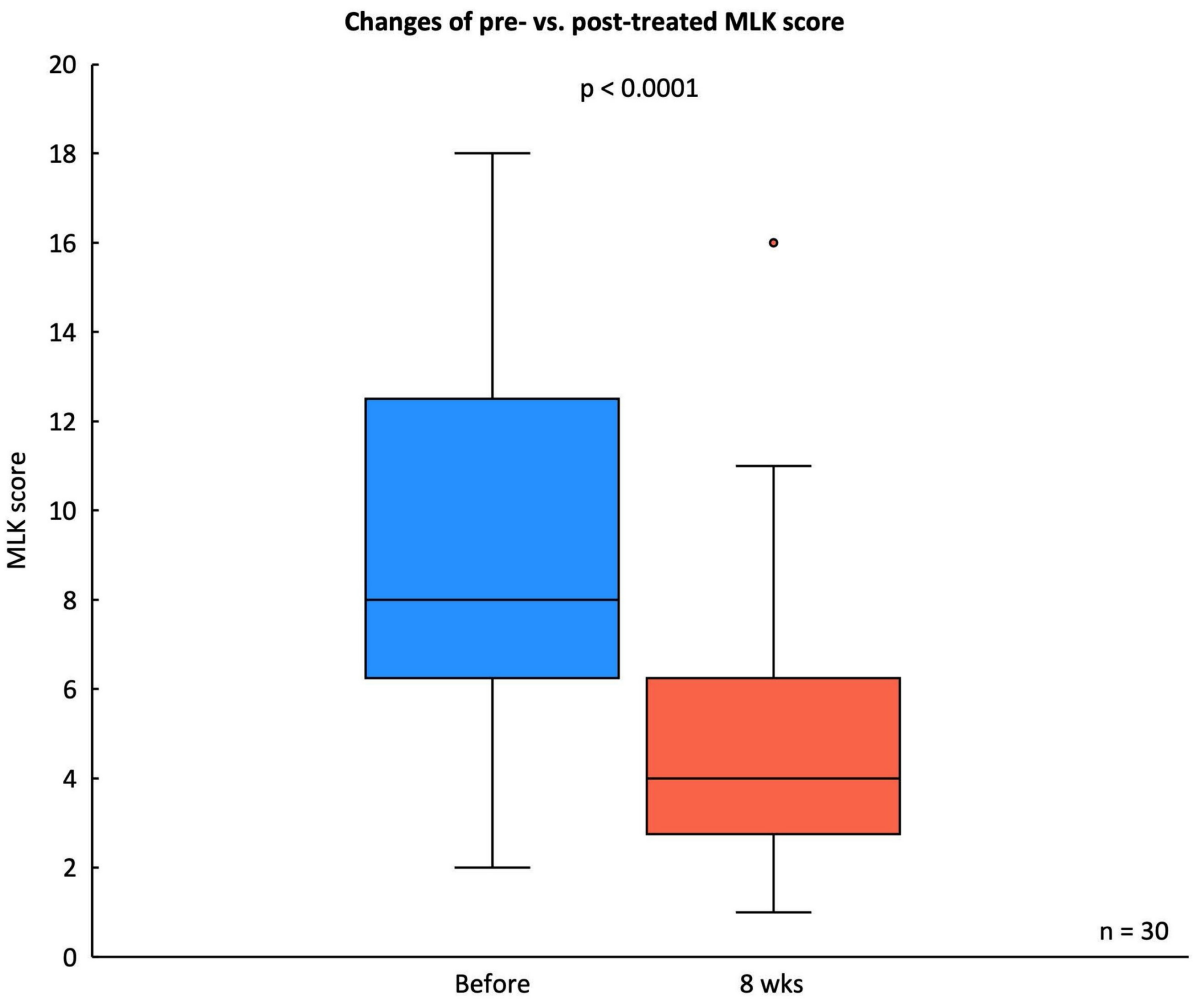
** Changes in MLK score before and after treatment.** Box plot showing the MLK scores from endoscopic evaluation in 30 CRSwNP patients before and after 8 weeks of *G. glabra* nasal spray treatment. Statistical significance is indicated with p < 0.0001.

The MLK endoscopic score decreased significantly from a baseline median score of 9.16 ± 4.44 to a post-treatment score of 5.03 ± 3.34 after eight weeks of *G. glabra* nasal spray treatment (p < 0.001), indicating substantial improvement in mucosal edema, discharge, and nasal patency (Fig. 5).

**Figure 6:**
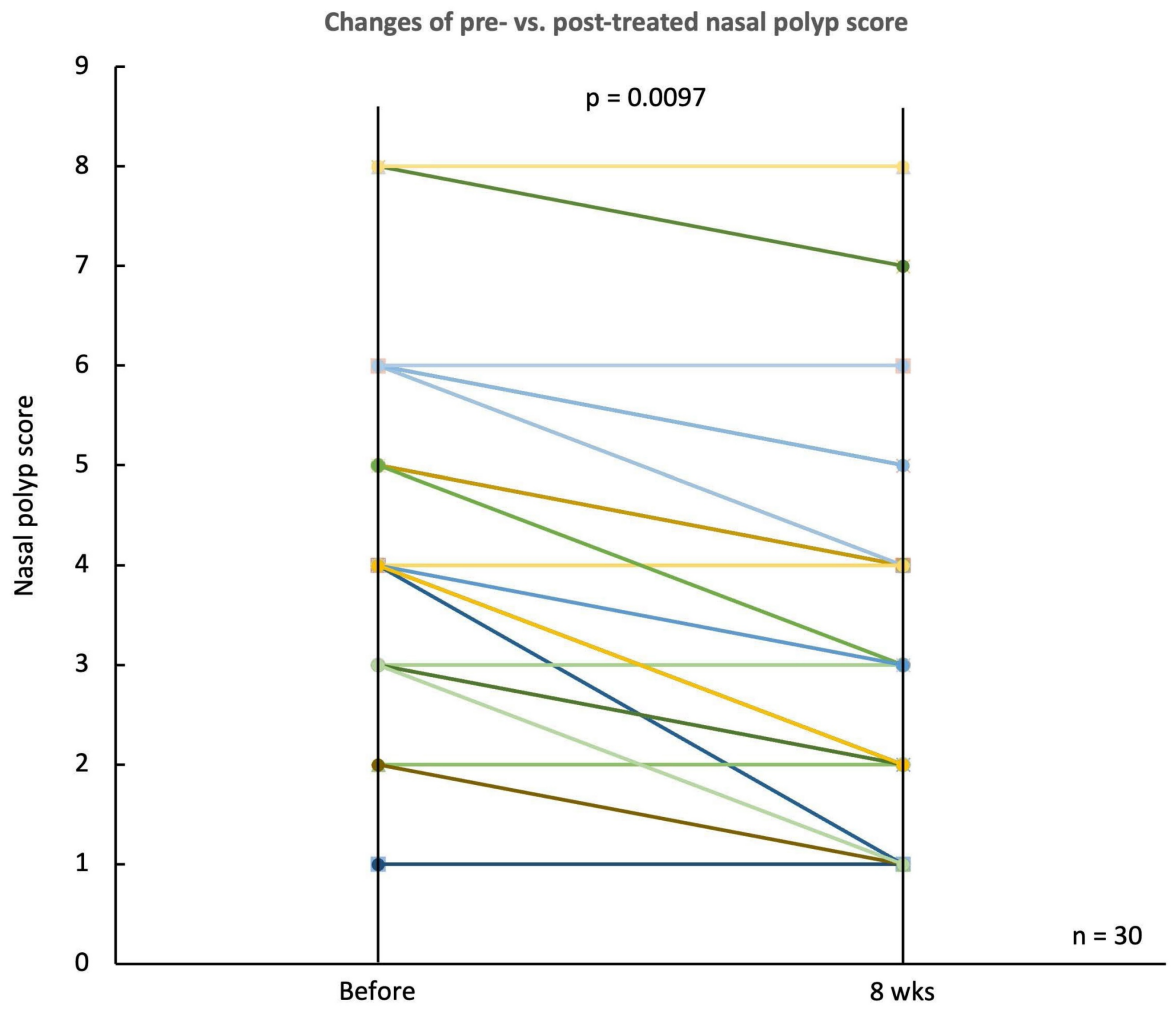
** Changes in nasal polyp score before and after treatment.** Line plot illustrating the nasal polyp scores for 30 CRSwNP patients before and after 8 weeks of treatment with *G. glabra* nasal spray. Each line represents an individual patient, showing the trend of polyp size reduction post-treatment. The overall decrease in nasal polyp scores is statistically significant, with p = 0.0097, suggesting an improvement in nasal polyp severity following the intervention.

Similarly, the nasal polyp score, depicted in a sloped line graph, demonstrated a notable decline from a baseline median score of 4.30 ± 1.62 to 3.13 ± 1.35 at the end of the treatment period (p = 0.0097) (Fig. 6). This decrease in nasal polyp score reflects a reduction in polyp size and its extension into the nasal cavity.

### Histopathological Analysis of Nasal Polyps

**Figure 7:**
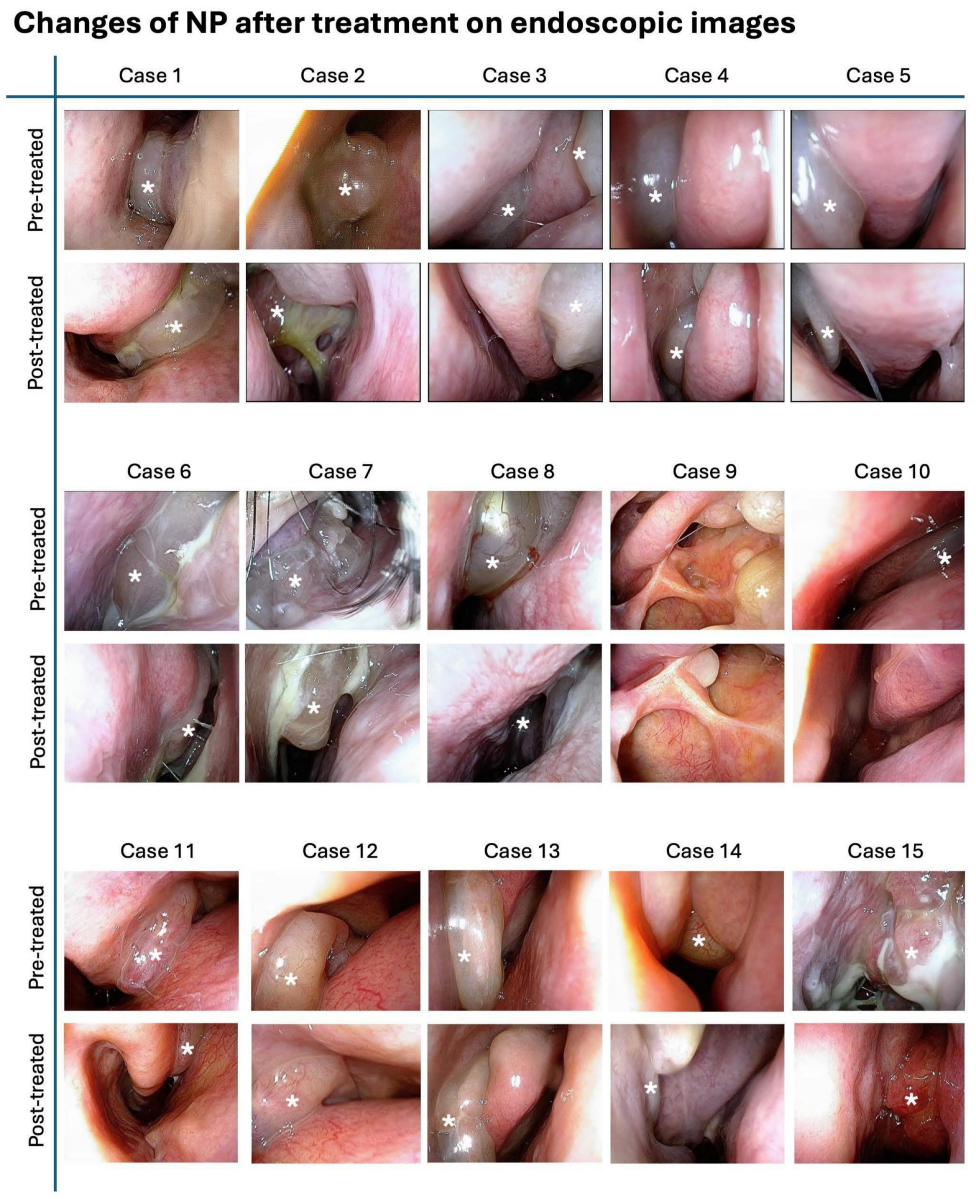
** Endoscopic images showing nasal polyp changes before and after treatment.** Representative endoscopic images of nasal polyps from 15 patients before and after treatment with *G. glabra* nasal spray. Each row of images displays the pre-treatment and post-treatment states of nasal polyps in individual cases. The white asterisks indicate the location of nasal polyps, showing reduction in polyp size and improvement in the nasal cavity condition following the intervention.

Figure 7 illustrates the endoscopic images of nasal polyps from 15 cases with CRSwNP who underwent *G. glabra* nasal spray treatment. These paired images, taken before and after the two-month treatment period, demonstrate a visible reduction in the size and extent of the polyps across the cases.

**Figure 8:**
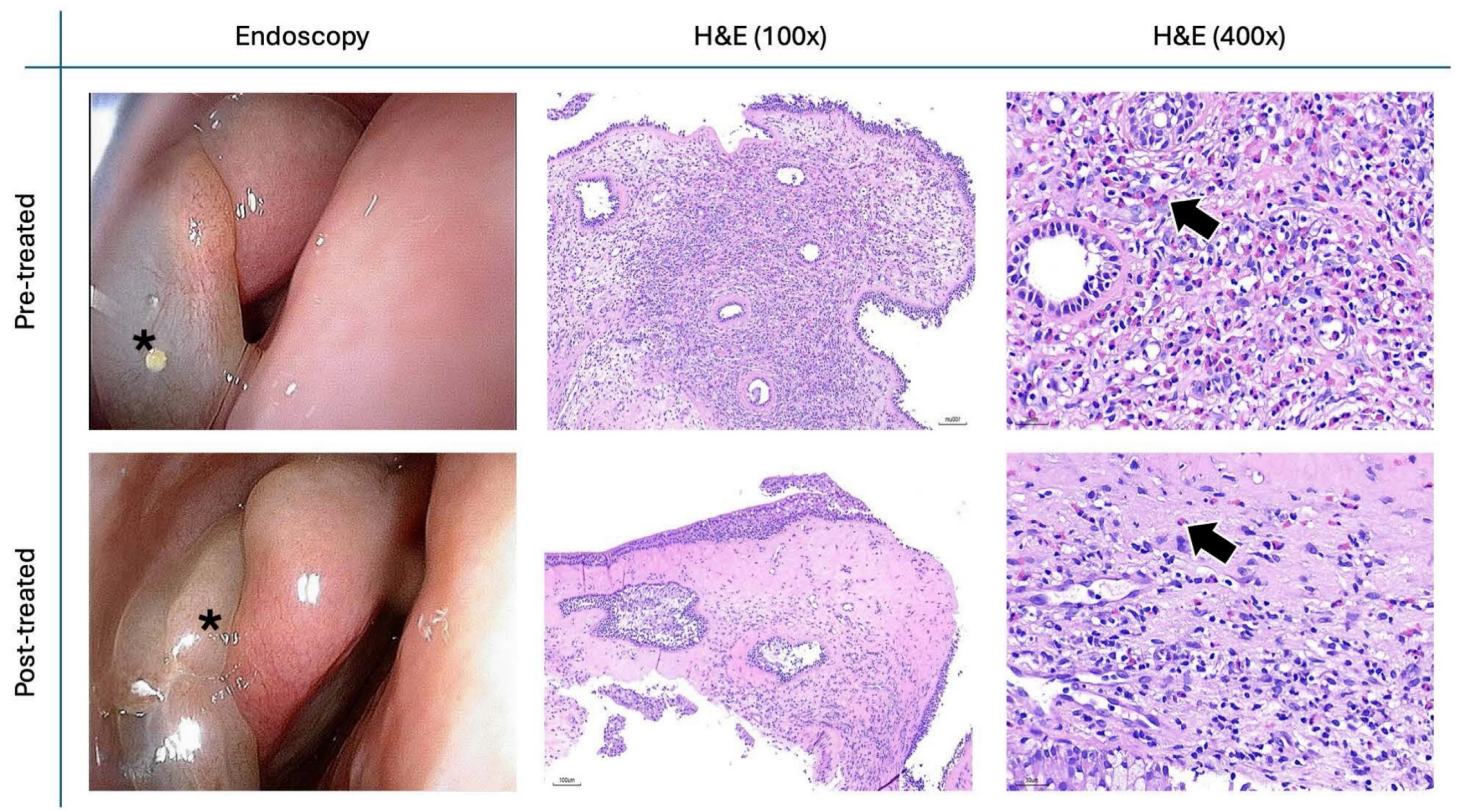
** Changes in nasal polyp H&E staining before and after treatment.** Endoscopic and histopathological images of a patient's nasal polyp before (top row) and after (bottom row) treatment. The black asterisk (*) marks the nasal polyp. H&E staining at 100x (middle column) and 400x (right column) magnifications shows a reduction in eosinophilic and lymphocyte infiltration post-treatment. Black arrows indicate eosinophils in the nasal polyp tissue.

Figure 8 presents representative pre- and post-treatment endoscopic and H&E-stained histological images from a CRSwNP patient treated with *G. glabra* nasal spray for two months. The H&E staining results demonstrate a significant reduction in eosinophil and lymphocyte infiltration within the nasal polyp tissue following treatment, indicating a decrease in inflammatory response.

**Figure 9:**
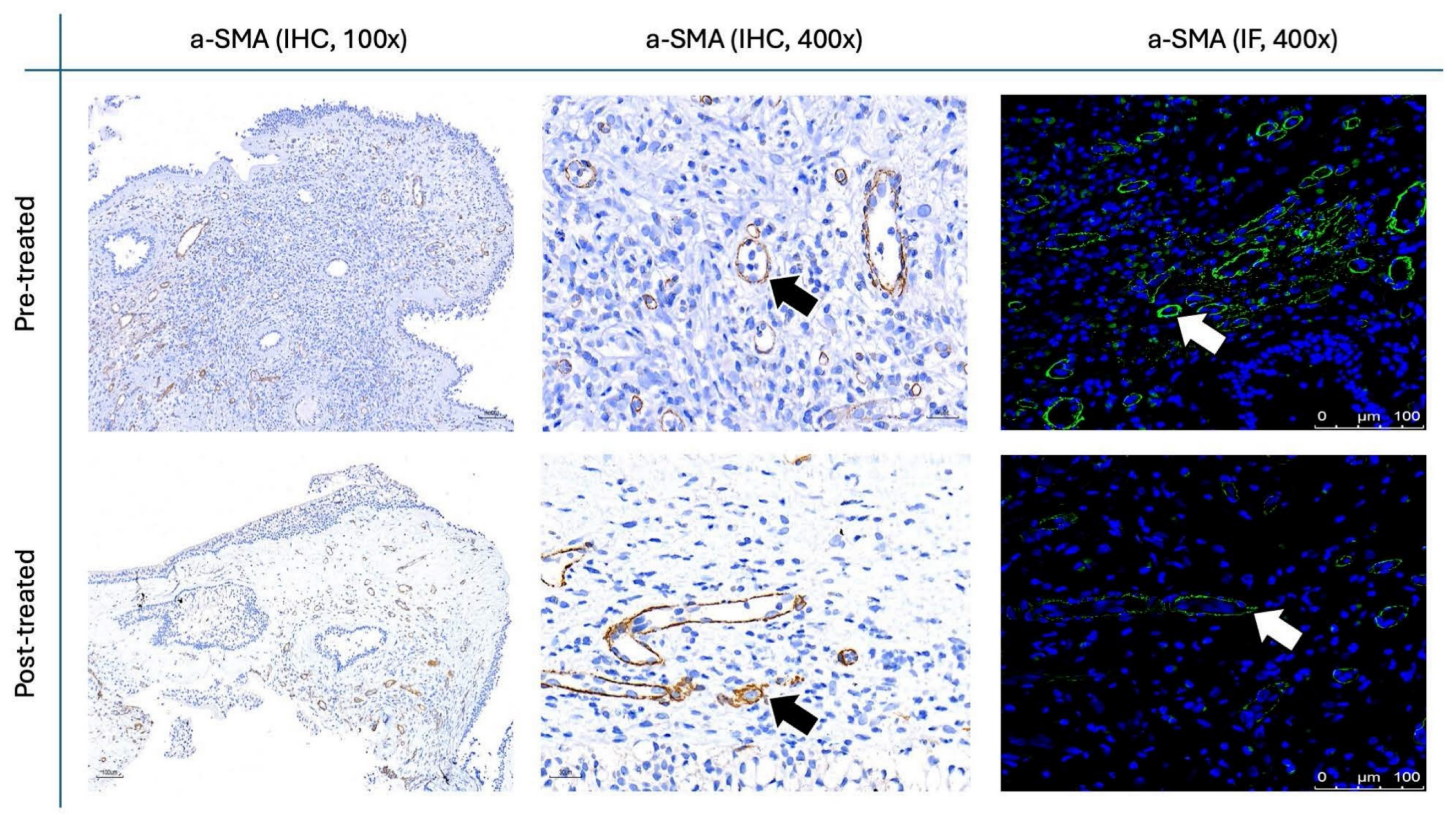
** α-SMA staining in nasal polyps before and after treatment with *G. glabra*.** Representative IHC and IF images showing α-SMA expression as an indicator of fibroblast activity. Images are shown at 100x and 400x magnification for IHC and 400x for IF, with black arrows indicating brown-stained areas in IHC and white arrows pointing to fluorescent-stained areas in IF.

**Figure 10:**
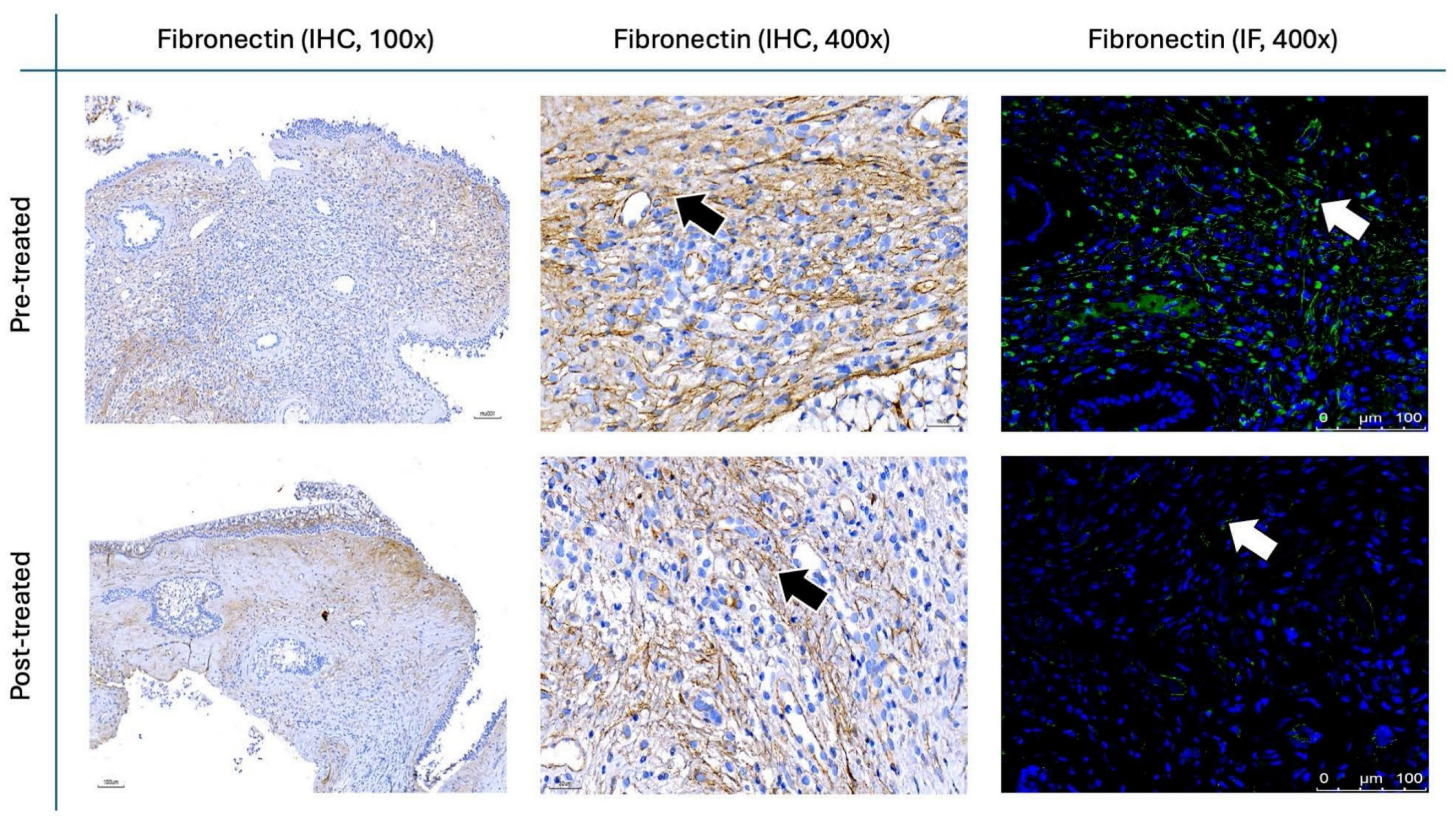
** Fibronectin staining in nasal polyps before and after treatment with** ***G. glabra*****.** Representative IHC and IF images demonstrating fibronectin expression. IHC images are shown at 100× and 400× magnifications, whereas IF images are presented at 400×. Brown-stained regions in IHC are indicated by black arrows, and fluorescent signals in IF are labeled with white arrows.

Figure 9 and Figure 10 illustrate the IHC and IF staining results for α-SMA and fibronectin, respectively. Following the two-month treatment with the *G. glabra* nasal spray, α-SMA expression demonstrated a marked reduction in staining intensity, suggesting a significant decrease in fibroblast activity. Additionally, fibronectin, an essential component of the ECM involved in structural support, exhibited a substantial decrease in both IHC and IF post-treatment.

**Figure 11:**
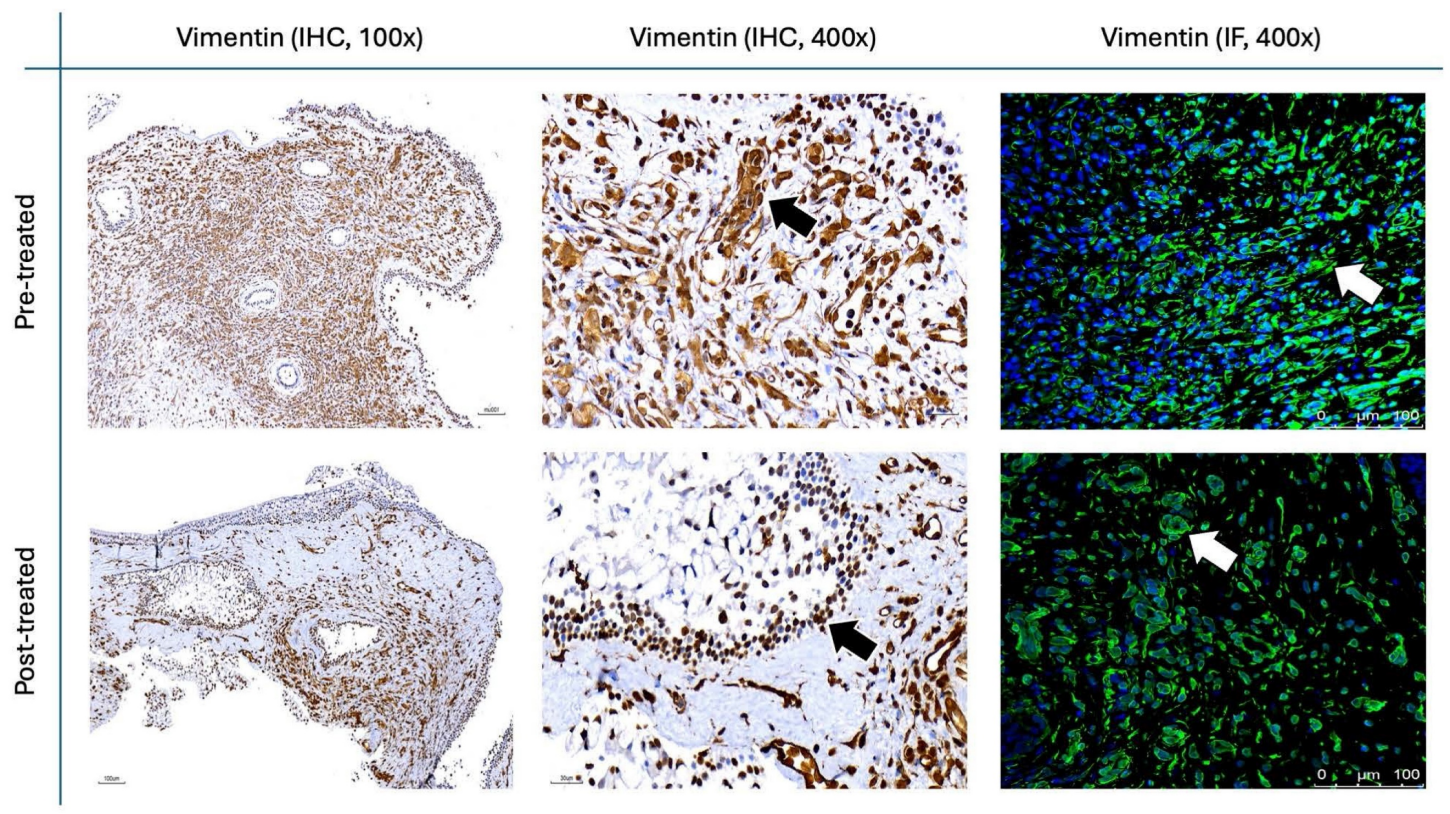
** Vimentin staining in nasal polyps before and after treatment with** ***G. glabra*****.** Representative IHC and IF images illustrating vimentin expression as a mesenchymal marker. Representative IHC micrographs were captured at 100× and 400×, and IF images were obtained at 400× magnification. Black arrows mark positive IHC staining, while white arrows highlight fluorescence in IF.

**Figure 12:**
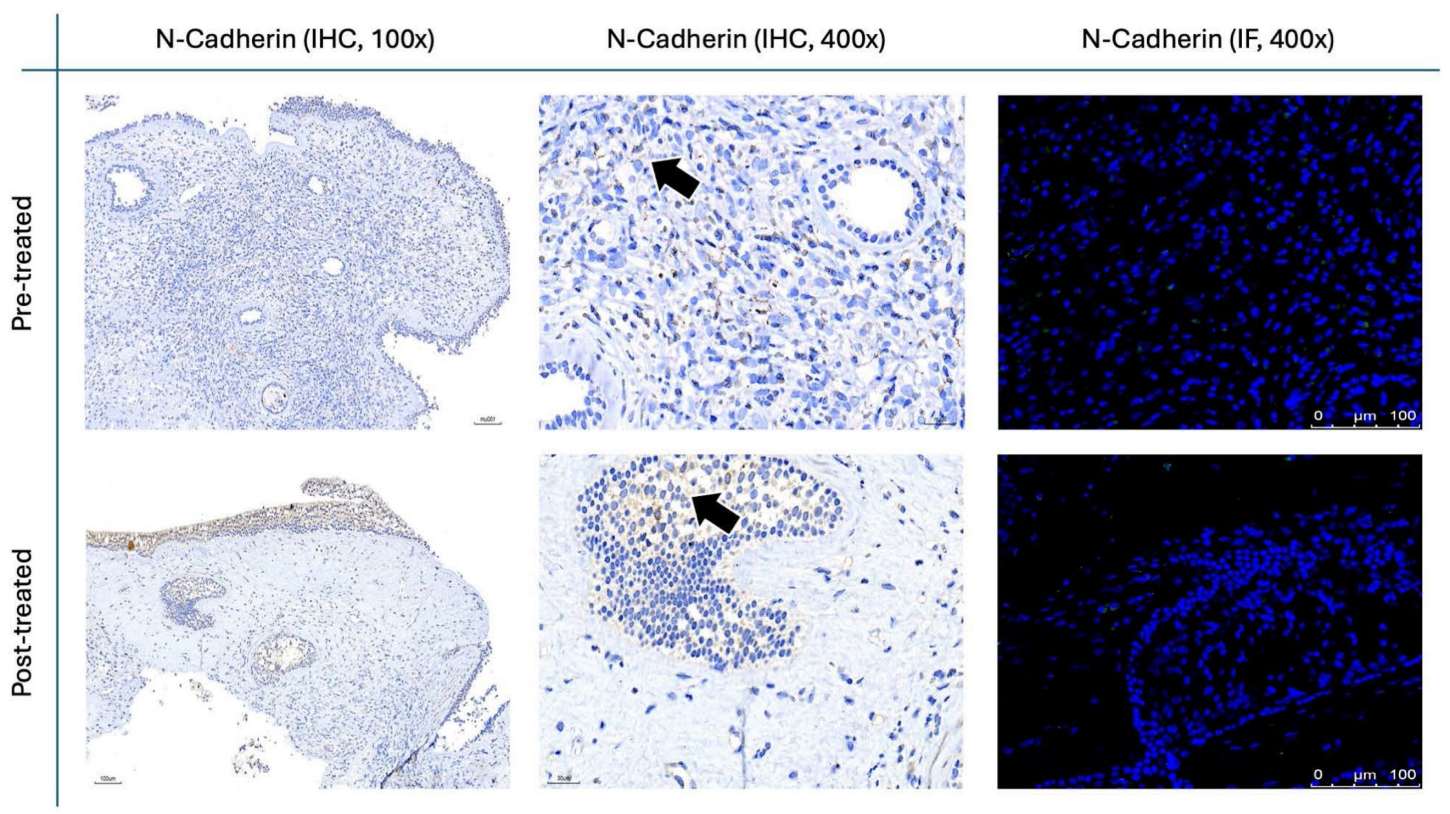
** N-cadherin staining in nasal polyps before and after treatment with** ***G. glabra*****.** Representative IHC and IF images showing N-cadherin expression. For IHC, images are displayed at both low (100×) and high (400×) magnifications; IF images are shown at 400×. Black arrows denote immunoreactive areas in IHC.

**Figure 13:**
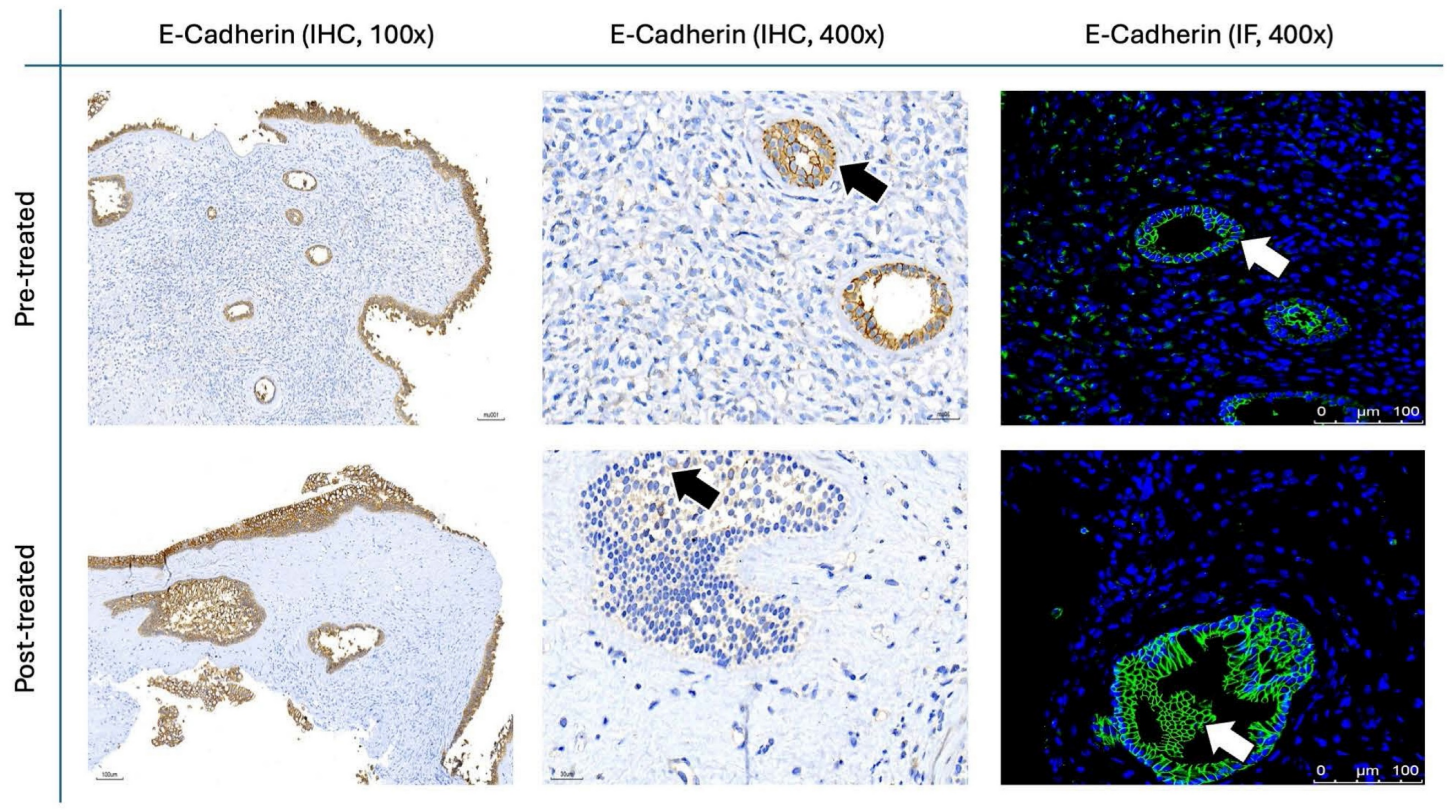
** E-cadherin staining in nasal polyps before and after treatment with** ***G. glabra*****.** Representative IHC and IF images demonstrating E-cadherin expression. Images for IHC and IF were acquired at standard magnifications (100× and 400× for IHC; 400× for IF). Chromogenic staining in IHC is indicated by black arrows, and fluorescence in IF is shown by white arrows.

**Figure 14:**
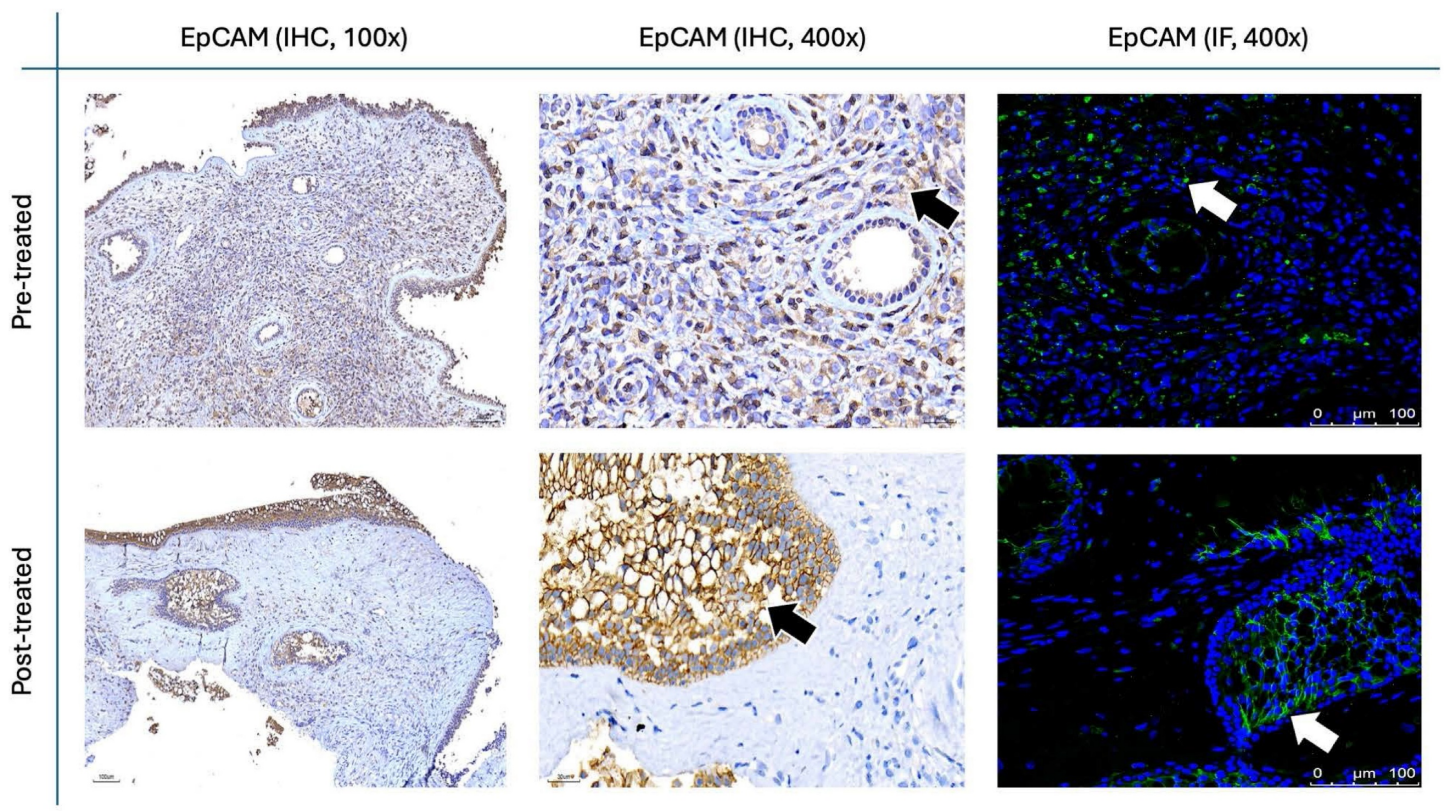
** EpCAM staining in nasal polyps before and after treatment with** ***G. glabra*****.** Representative IHC and IF images showing EpCAM expression. IHC panels are presented at 100× and 400× magnifications, while IF panels are shown at 400×. Positive staining appears as brown signals marked by black arrows in IHC and fluorescent signals indicated by white arrows in IF.

The results for Figure 11 through Figure 14 show the changes in epithelial and mesenchymal markers pre- and post-treatment. In Figure 11, vimentin, a mesenchymal marker, displayed a significant reduction in staining intensity after treatment, indicating a decrease in mesenchymal characteristics in nasal polyps. In contrast, the epithelial markers E-cadherin (Fig. 13) and EpCAM (Fig. 14) showed an increase in staining, suggesting a restoration of epithelial properties post-treatment. In Figure 12, N-cadherin staining presented a low proportion and weak intensity in both IHC and IF analyses, making it challenging to observe any substantial changes pre- and post-treatment.

**Figure 15:**
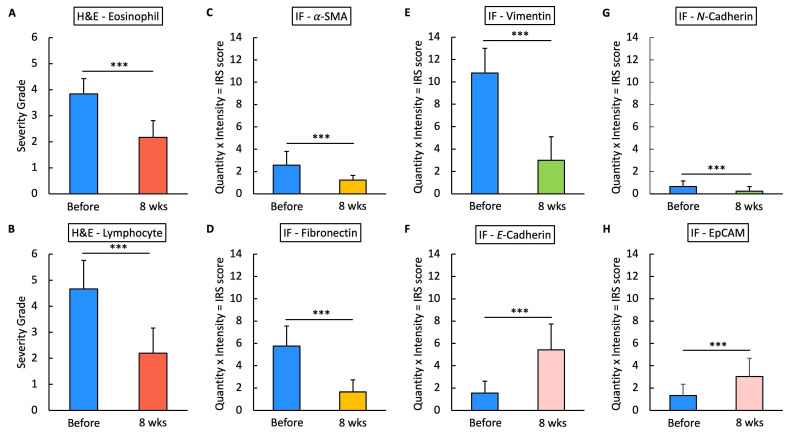
** Statistical analysis of H&E staining and IF IRS scores.** Fig.15 presents the statistical analysis results of H&E staining and IF immunoreactivity scores (IRS) for various markers in nasal polyp tissues before and after 8 weeks of treatment. The IRS score is calculated as the product of the quantity and intensity scores for each marker. Panels A and B show the severity grades of eosinophils and lymphocytes based on H&E staining, while panels C through H illustrate IRS scores for markers α-SMA, fibronectin, vimentin, E-Cadherin, N-Cadherin, and EpCAM in IF. Triple asterisks (***) indicate a statistically significant reduction or increase with p < 0.0001.

For the histopathological analysis (Fig. 15), we utilized the INHAND five-tier grading system 24 to assess eosinophil and lymphocyte infiltration on H&E stain (Fig. 8), while IF intensity was quantified using the IRS system for various biomarkers 25. In eosinophil and lymphocyte counts, the H&E scores significantly decreased after 8 weeks of* G. glabra* nasal spray treatment (scores: 3.83 ± 0.59 before vs. 2.16 ± 0.64 after for eosinophils, 4.66 ± 1.09 before vs. 2.20 ± 0.96 after for lymphocytes, p < 0.0001). For IF, markers of fibroblast activity (α-SMA, Fig. 9) and ECM production (fibronectin, Fig. 10) also showed notable reductions post-treatment (α-SMA scores: 2.56 ± 1.25 before vs. 1.23 ± 0.43 after; fibronectin scores: 5.76 ± 1.79 before vs. 1.66 ± 1.06 after, p < 0.0001).

Vimentin staining (Fig. 11) demonstrated a marked decrease in mesenchymal expression (scores: 10.80 ± 2.20 before vs. 3.00 ± 2.10 after, p < 0.0001). Conversely, epithelial markers E-cadherin (Fig. 13) and EpCAM (Fig. 14) showed increased expression following treatment (E-cadherin scores: 1.56 ± 1.04 before vs. 5.43 ± 2.29 after; EpCAM scores: 1.33 ± 0.99 before vs. 3.03 ± 1.62 after, p < 0.0001), indicating an epithelial recovery. Although N-cadherin staining in IF was relatively weak with limited staining coverage, statistical analysis revealed a significant decrease in expression post-treatment (scores: 0.66 ± 0.49 before vs. 0.23 ± 0.43 after, p < 0.0001; Fig. 12).

## Discussion

In this clinical trial, we observed that the use of the *G. glabra* nasal spray in patients with CRSwNP led to significant improvements in both subjective symptoms and objective findings. Patients reported marked relief in their symptoms of post-treatment, and endoscopic examination revealed reduced signs of sinus inflammation and noticeable shrinkage of nasal polyps. Pathological examination of nasal polyp tissue further supported these clinical observations, showing a reversal in markers associated with EMT.

The graphical abstract illustrates the proposed mechanism underlying these changes. During the development of nasal polyps, EMT leads to a shift from epithelial characteristics to mesenchymal traits, characterized by a decrease in epithelial markers (E-cadherin and EpCAM) and an increase in mesenchymal markers (vimentin, FBN, and N-cadherin) 5-8. Our study found that after treatment with the *G. glabra* nasal spray, this EMT process was effectively reversed, with upregulation of epithelial markers and downregulation of mesenchymal markers. This reversal of EMT likely contributes to the observed improvements in nasal polyp size and symptom severity, suggesting that the *G. glabra* nasal spray may provide an effective non-surgical option for managing CRSwNP.

Given the heterogeneity in CRSwNP immunopathology, recent research has categorized the condition into endotypes based on distinct immune responses 26, 27: Type 1 (Th1-mediated, often associated with neutrophilic inflammation and IFN-γ), Type 2 (Th2-mediated, characterized by eosinophilic inflammation and elevated IL-4, IL-5, and IL-13), and Type 3 (Th17-mediated, related to IL-17 and neutrophil predominance). This immunologic classification not only enhances our understanding of CRSwNP pathogenesis but also supports a more personalized approach to treatment.

Our study has shown that *G. glabra* nasal spray may reduce symptoms and inflammation in CRSwNP patients, but it remains unclear whether its efficacy varies across these endotypes. Future studies could explore whether the anti-inflammatory effects observed are more pronounced in specific immune profiles, such as Type 2-dominated cases, which are most commonly associated with eosinophilic infiltration. Moreover, identifying the active compounds, including glycyrrhizin, liquiritin, isoliquiritin, liquiritigenin, et al 28, 29 in *G. glabra* that modulate fibroblast activity in nasal polyps could provide insights into the cellular mechanisms underlying its therapeutic effects. In addition, the licorice extract used in this study was prepared from *Glycyrrhiza glabra*, which was the only hospital-approved licorice material stocked by our TCM pharmacy during the study period. Future studies may explore potential differences among various licorice species commonly used in Asia, such as *Glycyrrhiza uralensis*.

This study has several limitations that should be addressed. First, the sample size was relatively small, with only 15 out of 30 CRSwNP patients completing pre- and post-treatment nasal polyp biopsies. This limited sample size may introduce statistical bias and affect the generalizability of our findings. Future studies with larger patient populations are recommended to validate these results and strengthen the statistical power.

Second, the treatment period in this study was restricted to eight weeks. While we observed significant improvements in subjective symptoms and endoscopic findings within this period, the long-term effects of G. glabra nasal spray on CRSwNP remain uncertain. Extending the treatment duration and follow-up period in future studies could provide a better understanding of the sustainability of the therapeutic effects and potentially uncover delayed responses that were not observable within the current timeframe. In addition, objective olfactory tests were not included in the present protocol. Future studies incorporating validated olfactory assessment tools and standardized testing procedures will help further clarify the treatment effect on olfactory function.

Lastly, this study focused solely on evaluating a single dosage and frequency of *G. glabra* nasal spray administration. Further research is needed to explore the optimal dosing regimen for maximizing efficacy while minimizing any potential adverse effects. Larger, multi-center randomized controlled trials could provide valuable insights into the ideal use of *G. glabra* nasal spray as a treatment for CRSwNP and help establish it as a standard therapeutic option.

## Conclusions

*G. glabra* nasal spray may serve as a novel, effective treatment for CRSwNP, offering notable improvements in symptom relief and reduction in nasal polyp size. The observed decreases in inflammatory cells and modulation of EMT markers highlight *G. glabra'*s potential to address both clinical and histopathological aspects of CRSwNP, positioning it as a promising non-surgical therapeutic option.

## Figures and Tables

**Table 1:** Demographic characteristics of cases with CRSwNP

Variables	CRSwNP (n = 30)	
Gender		
Male	24 (80%)	
Female	6 (20%)	
Age (Years)	46.1 ± 12.8^*^	
Pre-treated TNSS^a^	6.73 ± 3.03	
Pre-treated SNOT-22^b^	41.2 ± 19.7	
Pre-treated MLK score^c^	9.20 ± 4.44	
Pre-treated NP score^d^	4.30 ± 1.62	

Abbreviations: CRSwNP, chronic rhinosinusitis with nasal polyposis; a. TNSS, total nasal symptom score; b. SNOT-22, Sino-Nasal Outcome Test-22; c. MLK score, Modified Lund-Kennedy Score; d. NP score, nasal polyp score *. mean ± standard deviation

## Abbreviations

CRSwNP: Chronic rhinosinusitis with nasal polyps
ECM: extracellular matrix
EMT: epithelial-mesenchymal transition
GA: glycyrrhizic acid
*Glycyrrhiza glabra*: *G. glabra*: H&E: hematoxylin and eosin
HPLC: High-performance liquid chromatography
IF: immunofluorescence
IHC: immunohistochemistry
SNOT-22: Sinonasal Outcome Test-22
TNSS: Total Nasal Symptom Score
